# Deciphering the key stressors shaping the relative success of core mixoplankton across spatiotemporal scales

**DOI:** 10.1093/ismeco/ycaf053

**Published:** 2025-03-26

**Authors:** Zhicheng Ju, Sangwook Scott Lee, Jiawei Chen, Lixia Deng, Xiaodong Zhang, Zhimeng Xu, Hongbin Liu

**Affiliations:** Department of Ocean Science, The Hong Kong University of Science and Technology, Hong Kong SAR, 000000, China; Department of Ocean Science, The Hong Kong University of Science and Technology, Hong Kong SAR, 000000, China; Department of Ocean Science, The Hong Kong University of Science and Technology, Hong Kong SAR, 000000, China; Department of Ocean Science, The Hong Kong University of Science and Technology, Hong Kong SAR, 000000, China; Department of Ocean Science, The Hong Kong University of Science and Technology, Hong Kong SAR, 000000, China; Department of Ocean Science, The Hong Kong University of Science and Technology, Hong Kong SAR, 000000, China; Department of Ocean Science, The Hong Kong University of Science and Technology, Hong Kong SAR, 000000, China; Hong Kong Branch of Southern Marine Science and Engineering Guangdong Laboratory (Guangzhou), Hong Kong SAR, 510000, China

**Keywords:** mixoplankton, core taxa, key stressors, trophic modes, generalized additive mixed model, optimal niche

## Abstract

Deciphering the spatiotemporal dynamics and relative competitive advantages of trophic functional traits under multiple stressors has been a long-standing challenge. Here, we integrated the core taxa identification with robust simulation modeling to reveal key environmental factors influencing the three core trophic groups (autotroph, heterotroph, and mixotroph), with a particular focus on mixoplankton. Temporally, core mixoplankton exhibited a higher relative proportion in spring and winter in contrast to core heterotrophs and a more uniform spatial distribution pattern. While seasonal patterns were observed in the environmental responses of the trophic groups, temperature, dissolved oxygen (DO), and nitrate (NO_3_-N) were identified as the key drivers affecting the core mixoplankton by random forest. Furthermore, through univariate regression and generalized additive mixed model (GAMM), we captured the niche preferences of core mixoplankton across three stressors gradients and characterized the coupled additive or antagonistic effects. Notably, the potential optimal threshold for core mixoplankton was a high level of NO_3_-N (0.64 mg/L), lower temperature (18.6°C), and DO (3.5 mg/L), which contrasted with the results obtained from single-factor regression analyses. Specifically, GAMM indicated that the preferred niche shifted upward for NO_3_-N and downward for DO when three drivers were included simultaneously, while temperature remained constant. Our study linked the ecological niche preference of core mixoplankton with key stressors, facilitating a more precise monitoring and comprehension of spatiotemporal dynamics of trophic functional groups under scenarios of escalating global climate change and anthropogenic disturbances.

## Introduction

Marine plankton communities form the foundation of oceanic food webs and drive global biogeochemical cycles [1]. Over the past decade, the traditional dichotomy of classifying these organisms into photoautotrophs and phagoheterotrophs has gradually been abandoned [2]. Typically, planktonic protist communities are categorized into three main resource-harvesting groups based on trophic traits: autotroph, heterotroph, and mixotroph [3, 4]. In contrast to obligate strategies, mixoplankton integrate photosynthesis and phagotrophy within a cell, leading to dynamic competition among the trophic groups. This interspecies competition, driven by different trophic strategies, is crucial for shaping the relative success and environmental preferences of mixoplankton. However, it has been largely overlooked compared to the trophic trade-offs within the cell [5, 6]. While recent studies and reviews have highlighted the importance of mixoplankton in planktonic food webs and biogeochemical cycles, expanding our understanding of their distribution, abundance, and the conditions under which they thrive remain elusive [7, 8]. Investigating the spatiotemporal competitive dynamics of mixotrophic taxa and associated ecological determinants is recognized as a major priority [4, 9].

Coastal ecosystems are highly dynamic environments affected by climate change and human activities, where the three trophic groups are subjected to increasing compound pressures from multiple abiotic factors, such as temperature, nutrient availability, and dissolved oxygen (DO) [8, 10, 11]. Assessing and identifying the key factors driving the abundance and diversity among trophic groups is important for deciphering their niche differentiation and competitive advantages. However, this understanding is challenged by the high taxonomic diversity of plankton communities, sparse sampling, and the limitations of different research methodologies [4]. For instance, current experimental studies tend to conduct short-term controlled experiments with a few isolated strains [12, 13]. Additionally, methods estimating mixoplankton abundance and proportions through fluorescent labeling or microscopic examination have been shown to have unpredictable biases leading to under- or overestimation [12, 14]. More importantly, incorporating multiple stressors and the interaction typology (such as synergistic, additive or antagonistic) into considerations can enhance the explanatory and predictive power of current models, providing insights for ecological implications in changing environments [15, 16]. However, current studies predominantly focused on single factors or discussed their effects separately [17, 18], thereby obscuring the cumulative effects of multiple stressors commonly experienced under global change. Metabolic theory of ecology and previous research suggest that warming can not only directly influence growth and metabolic rates but may also indirectly shape the competitive dynamics among different trophic groups by reducing DO levels and nutrient concentrations [11, 18]. Hence, it is critical to consider the combined effects of multiple environmental pressures simultaneously.

High-throughput sequencing has provided opportunities to reveal the diversity, competitive dynamics, and environmental drivers of trophic groups at the community level [4, 19]. However, the primary challenge is how to annotate the vast number of species present in sequencing data with their corresponding trophic functional traits. This task is complicated by limited availability of cultured taxa and databases of mixoplankton [9]. For example, Faure *et al.* [6] identified only 133 mixotroph lineages out of 5071 amplicon sequence variants (ASVs) based on sequencing data from 659 samples in the Tara Oceans project. While it is encouraging that the Mixoplankton Database has been integrated into PR2 [9], laying the groundwork for trophic annotations, identifying core taxa may offer a shortcut to addressing this challenge. Studies have shown that discovering a core microbiome remains a key objective that helps inform the stable and consistent components across dynamic microbial community [20, 21]. Baquerizo *et al.* [22] found that a small number of core species can contribute to more than half of the community abundance and are closely linked to functional clusters. These core taxa tend to be more responsive and adaptable to environmental changes, making them effective indicators for tracking community succession and spatiotemporal shifts [23]. The approach allows researchers to narrow down the immense number of taxa to a “most wanted” list, thereby obtaining a higher proportion of annotations without requiring extra extensive knowledge. One way to systematically explore the abundance and occupancy thresholds used to define the core microbiome is to evaluate how well the resulting core membership reflects the overarching patterns of the full dataset [20, 21]. Accordingly, Shade *et al.* [21] proposed a generalized method that considered both temporal and spatial factors and integrated the contribution of β diversity, which is expected to be suitable for defining core marine plankton taxa. Moreover, ecosystems typically do not respond to multiple stressors smoothly and linearly. Instead, the community may reach the optimal ecological niche under the cumulative effects, such as synergism or antagonism [24]. Random forest (RF) model and generalized additive mixed model (GAMM) have emerged as two of the most promising methods complementing each other in identifying and modeling key environmental stressors [25, 26]. By processing multiple variables and evaluating their relative importance, RF effectively screens and ranks the main factors influencing microbial community structure, providing critical quantitative support for understanding the driving mechanisms [27, 28]. In contrast, GAMM further offers precise analysis of nonlinear and complex interactions in modeling the key factors identified by RF, without relying on pre-assumed relationships between variables [26, 29]. These methods enhance the accuracy in capturing the interplays and optimal thresholds across the gradiences of stressors on ecosystems.

In this study, we combined observed data and predictions from Kriging interpolation, RF model and GAMM to decipher the spatiotemporal distribution patterns of core taxa, particularly mixoplankton, and identify the key stressors shaping their competitive landscape. We proposed addressing the following scientific questions: (i) How do the abundance and diversity of three core trophic groups vary and distribute across spatiotemporal scales? (ii) Do they exhibit preferences in environmental responses, and are there key factors that consistently drive their dynamic coexistence? (iii) How do these key stressors shape the relative success of core mixoplankton, and is it possible to identify its niche optimum?

## Materials and methods

### Study sites and environmental factors

**Figure 1:**
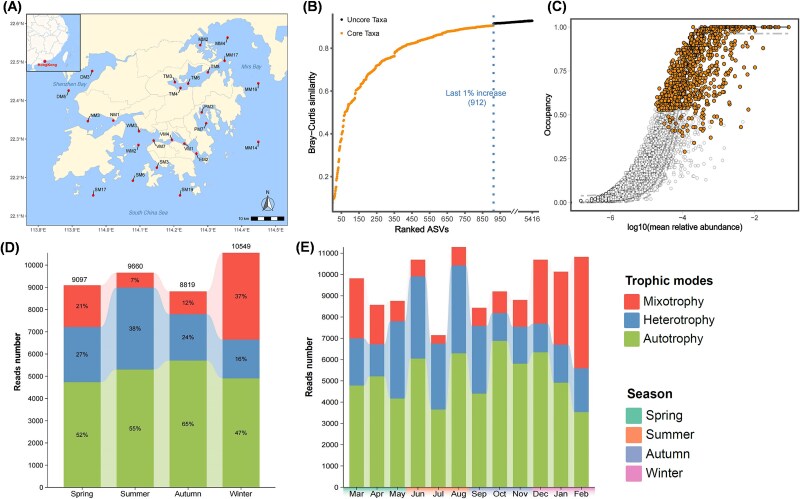
Definition of core trophic taxa and dynamics of their relative abundance. (A) Geographical distribution of 24 sampling sites around Hong Kong waters, south of China. (B) The contribution of the top-ranked taxa is divided by the total Bray–Curtis similarity to calculate a percent contribution of the prospective core set to beta diversity. The vertical dotted line distinguishes core taxa (912 ASVs) by the last 1% increase in explanatory value by Bray–Curtis similarity. (C) Neutral model fits to abundance-occupancy distributions. Each point is an ASV plotted by its mean log10 relative abundance and occupancy in the neutral model with 95% confidence intervals around the model fit. Absolute abundance (reads) and their relative proportions of three trophic core taxa within seasons (D) and months (E).

The waters surrounding Hong Kong, a subtropical coastal region frequently subjected to anthropogenic disturbances and water exchange, represent a typical area for studying the ecological consequences of multiple environmental stressors. Over the past three decades, this region has experienced numerous episodes of aquatic ecosystem degradation and recovery [30]. To minimize spatiotemporal biases and ensure the observed patterns reflect ecological realities, we conducted high-density spatial and monthly sampling at 24 stations along Hong Kong coast (Fig. 1A) and collected surface seawater samples (~0.5 m). About 500 mL of water sample was filtered through 0.22 μm polycarbonate filters. In addition, the corresponding geographic and environmental data for each sample were obtained from the Environmental Protection Department of Hong Kong government. Details for measuring these factors have previously been described [25]. Geographic data included latitude and longitude. The 14 environmental factors were divided into two categories: Nutrient and Physicochemistry. The former included total nitrogen (TN), nitrate (NO_3_-N), ammonium (NH_4_-N); total phosphorus (TP), phosphate-phosphorus (PO_4_-P) and TN: TP ratio (NP ratio). The latter included temperature, salinity, turbidity, pH, Chlorophyll a, DO, suspended solids (SS) and Secchi.

### 18S rRNA gene metabarcoding

DNA was extracted from the filters using the FastDNA SPIN Kit (MP Biomedicals, Santa Ana, CA, USA) and was amplified using the 18S rRNA universal primer set, 528F forward (5′-GCGGTAATTCCAGCTCCAA-3′) and 706R reverse (5′-AATCCRAGAATTTCACCTCT-3′), that targeted the V4 hypervariable regions. It has been proven effective for amplifying a broad range of eukaryotic taxa, ensuring that we captured the representative the planktonic community. The polymerase chain reaction (PCR) amplification included initial denaturation at 94°C for 5 min, followed by 35 cycles at 94°C for 1 min, 48°C for 2 min, and 72°C at 1 min, and final extension 5 min at 72°C. PCR products from each sample were purified and pooled in equal amounts, followed by pair-end sequencing using the Illumina NovaSeq platform.

### Bioinformatics and statistical analysis

The raw sequences were analyzed according to the revised EasyAmplicon pipeline v1.20 [31]. Briefly, paired-end reads firstly were merged, primer-removed, quality-controlled, and dereplicated. Subsequently, ASVs were clustered using Unoise3 in USEARCH v10. ASVs with fewer than 8 sequences were removed to minimize sequencing errors. Finally, all samples were rarefied to the same sequencing depth for downstream analysis. Representative sequences were assigned to taxonomic lineages via Protist Ribosomal Reference (PR2) database (v5.0.0) [32].

#### Definition of the core subset and its trophic annotation

To incorporate heterogeneity caused by spatial/cross-sectional consideration and minimize biases from rare species, we followed the method developed by Shade et.al [21] to identify core plankton trophic groups. The approach first ranked the ASVs by occupancy across temporal and spatial scales and then calculated the minimal prevalence threshold dynamically. The proportion of total community explained by core subset estimated using the Bray-Curtis distance for β diversity, and core-taxa identified at 1% as the threshold for a marginal return in the explanatory value.

In order to assign as many ASVs as possible to the three trophic modes, we utilized the combined methods including synthesized published databases/sets and manual searches based on the latest research [6, 12, 32, 33] (Table S1). Most of the annotations for the potential mixoplankton were taken from the column “mixoplankton” in PR2 database using a custom script. Please note that when there are discrepancies between PR2 database information and the latest literatures, we prioritized the latter due to its clearer cultivation evidence and interpretation, as well as the potential delays in database updates. Unknown taxa or those with unavailable trophic evidence were all assigned as others/unknown and excluded from subsequent analysis [12].

#### Kriging interpolation mapping

We applied a kriging interpolation method to map and predict the distribution of Shannon diversity of three core trophic groups across Hong Kong waters. The geographic coordinates of sampling sites and Shannon diversity indices were merged, and ordinary kriging was performed using the “automap” package in R 4.2.3, which automates the interpolation by estimating a semivariogram and performing kriging [34]. Cross-validation of the predictions was conducted using Pearson’s correlation between the predicted and observed values at each sampling site to ensure the model’s reliability and accuracy.

#### Random forest model

RF is one of the most robust ensemble machine learning classifiers with excellent accuracy among current algorithms for classification and regression [35]. It is gaining increasing attention in ecological studies due to its ability to effectively handle complex non-linear relationships and identify key predictive variables [27, 28]. The rfPermute package was utilized for the ranking and estimation of the importance and significance of variables. In RF models, we included 14 environmental parameters that were predictors by calculating the precision importance of each tree and averaging over the forest (1000 trees). The percentage increase in mean square error (MSE) of the variables was used to estimate the importance of these predictors, with higher MSE% values implying more important predictors.

#### Generalized additive mixed model

GAMM was employed to investigate the combined effects of three key factors identified by RF utilizing the mgcv package in R. All samples were converted into factor variables, and extreme outliers (the z-score greater than 3) were removed. Logarithmic transformation was applied to the relative proportion to normalize the data distribution. To address multicollinearity concerns, we computed variance inflation factors (VIF) for each predictor to ensure independence among variables (Fig. S5). GAMM was conducted iteratively, starting with a base model and progressively introducing interaction terms to capture combined effects, and we developed four hierarchical GAMMs in total. The model equations, explanations and reliability were detailed in the supplementary material.

The combined effects and interactions modeled by GAMM were visualized through 2D and 3D plots. The potential optimal thresholds for core mixoplankton were identified by examining the inflection points in the smooth functions from the plots [36, 37]. It corresponded to the conditions under which the interaction of the three main drivers resulted in the maximum response of core mixotroph reads. All models were fitted using the Gaussian family, and model comparisons were conducted based on Akaike Information Criterion (AIC) values (Table S3). The model with the lowest AIC was selected as the final model, balancing model complexity with goodness-of-fit. The significance of smooth terms and interaction effects was evaluated to interpret the impact of environmental variables on microbial community composition. Random effects accounted for spatial variability across sampling stations, ensuring robust estimates (Figs S5 and S6).

## Results

### Three core trophic plankton groups and their seasonal dynamics

The seasonal dynamics of environmental variables are presented in Fig. S1. Most parameters did not show a consistent pattern across locations and season scales. Instead, the values in different sites generally showed obvious but sudden peak or trough across seasons, indicating that these parameters in Hong Kong waters were highly disturbed. This highly dynamic variation in environmental heterogeneity within and between spatial and temporal scales would lead to potential multiple environmental stressors for diverse trophic communities.

In total, 5416 ASVs were obtained among the samples after rarefaction. We then identify a core group (912 ASVs) that accounted for 16.8% of total ASVs but contributed more than 80% of β diversity to the whole community (Figs 1B and C and S2). Furthermore, over 63% of core ASVs were annotated at the genus or species level, with autotroph, heterotroph, and mixotroph accounting for 54%, 24.6%, and 21.4%, respectively (Fig. S2). At the seasonal scale, the relative proportion of core autotrophs, accounting for about half of the reads abundance, generally showed less variation (Fig. 1D). In contrast, the abundances of core mixoplankton and heterotrophs exhibited significant seasonal variations (Fig. 1D). Core mixoplankton displayed a gradual increase from summer to winter and peaking in winter, while heterotrophs showed an opposite pattern. Specifically, core mixotrophic taxa exhibited a distinct seasonal pattern characterized by higher abundances in spring and winter (21%–37%) and lower abundances in summer and autumn (7%–12%), with a dominance in February, accounting for nearly half (48.4%) of the total abundance (Fig. 1D and E).

### Characteristics of diversity patterns on spatiotemporal scales

**Figure 2:**
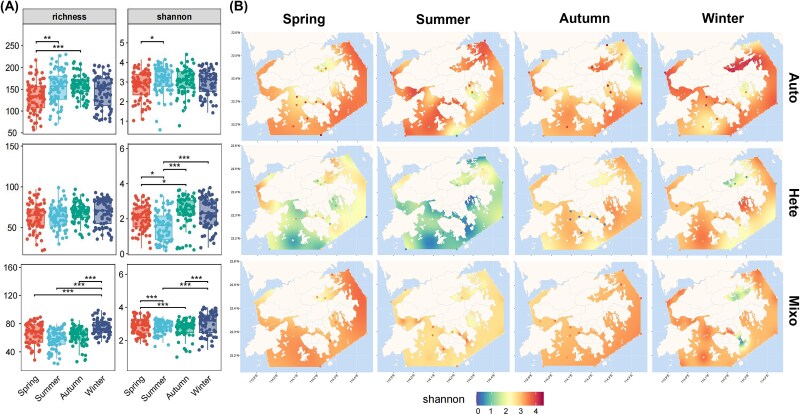
T temporal and spatial distribution discrepancy of α-diversity among three core trophic groups. (A) Seasonal differences in α-diversity (richness and Shannon). ^*^^*^^*^indicates *P* < .001; ^*^^*^indicates *P* < .01; ^*^indicates *P* < .05. (B) Predicted spatiotemporal Shannon distribution of core trophic taxa α-diversity using the co-kriging interpolation method. Auto: autotroph; Hete: heterotroph; Mixo, mixotroph.

**Figure 3:**
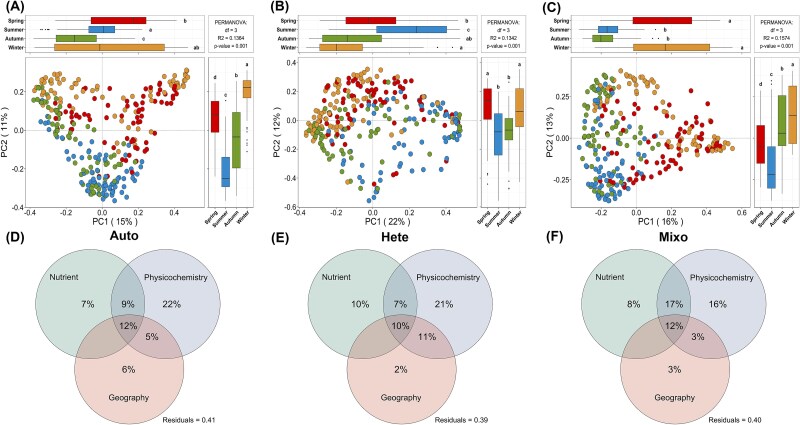
Seasonal consistent pattern of β diversity and quantifying the contribution of three kinds of environmental drivers. (A–C) the results of the unconstrained PCoA were visualized in a scatter plot, with samples color-coded by season. The first two principal components (PC1 and PC2) were plotted, with their respective explained variances. PERMANOVA with 999 permutations and pairwise comparisons were used to test for significant differences in community composition among seasons. (D–F) the relative contributions (%) of three kinds of environmental categories (nutrient, physicochemistry, and geography) are identified via VPA. Nutrient: TN, nitrate (NO_3_-N), ammonium (NH_4_-N), TP and phosphate phosphorus (PO_4_-P). Physicochemistry: temperature, salinity, turbidity, Secchi depth, Chlorophyll a, pH, DO and SS. Geography: longitude and latitude.

We treated the groups within the same core trophic type as a whole to investigate the seasonal variations of community diversity across three trophic taxa (Figs 2A and 3). Meanwhile, we mapped the spatial distributions with Shannon index accordingly using a kriging interpolation method (Fig. 2B; Fig. S3). Core autotrophs showed slight seasonal dynamics in reads abundance and α-diversity, yet maintained the higher local Shannon diversities, especially in some areas near the inner Bay (Fig. 2B). Although core heterotrophs displayed a seasonal pattern in reads abundance, no significant changes in richness were observed (Fig. 1D; Fig. 2A). In contrast, the core mixoplankton exhibited a more uniform spatial distribution and the most consistent “U-shaped” seasonal pattern, maintaining higher levels in spring and winter among relative proportion, richness and Shannon and reaching a peak in winter (Figs 1D and 2). In addition, the results of unconstrained principal coordinates analysis (PCoA) and permutational multivariate analysis of variance (PERMANOVA) test showed that the three core trophic groups shared a relatively consistent β-diversity pattern (Fig. 3A–C). Although significant differences (*P* < .001) in community composition were generally observed at the seasonal scale, the clusters tended to be more similar during the winter–spring and summer-autumn periods (Fig. 3A–C). This was consistent with the observed variation in corresponding reads abundance (Fig. 1D and E).

### Quantification of environmental variable impacts and identification of key factors

**Figure 4:**
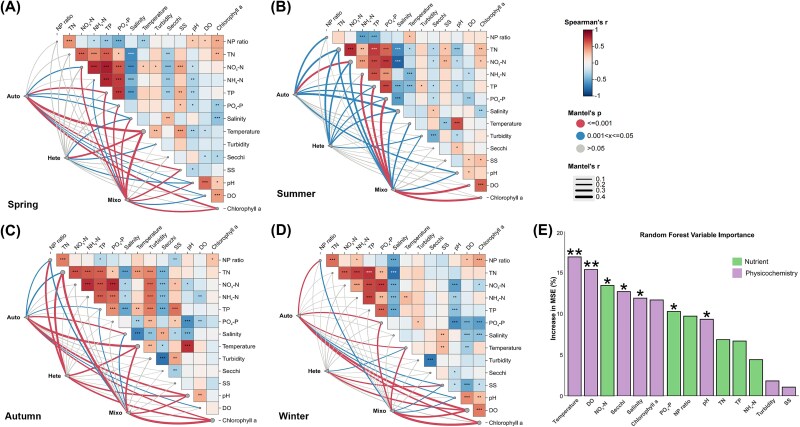
Mantel test and RF algorithm are used to identify the key environmental stressors. (A) Pairwise comparisons of 14 environmental factors are shown at the upper-right with Spearman's correlation coefficients. Three trophic groups (Auto: autotroph; Hete: heterotroph; Mixo, mixotroph) are correlated to each factor by partial mantel test. The line width represents the partial Mantel's *r* statistic for the corresponding correlation, and significances are tested based on 999 permutations. NP ratio, total nitrogen: total phosphorus ratio; TN, total nitrogen; NO_3_-N, nitrate; NH_4_-N, ammonium; TP, total phosphorus; PO_4_-P, phosphate phosphorus; temperature; salinity; SS, suspended solids; turbidity; Secchi; pH; DO, dissolved oxygen; Chlorophyll a. (B) Ranking mean predictor importance (percentage of increase of MSE) of environmental factors by RF model. The ranking of variable importance was visualized through the bar chart, which distinguishes nutrient and Physicochemistry groups in purple and green, respectively. MSE, mean squared error. Significance levels: ^*^P < .05, ^*^^*^P < .01.

To identify the key environmental factors influencing the abundance of core mixotrophs, we further explored how the parameters shape the relative competitive advantage of three nutritional types across the spatial and temporal scales. Since most factors varied dramatically over seasons and locations (Fig. S1), we first divided the 14 parameters into three categories (Nutrient, Physicochemistry and Geography) and then utilized the variance partitioning analysis (VPA) to quantify their relative influences (Fig. 3D–F; Table S2). In general, these categories exhibited strong interactive effects. However, the consistent influence ranking on the three core trophic groups was as follows: Physicochemistry (16%–22%) > Nutrient (7%–10%) > Geography (2%–6%). Furthermore, the impact of the first two environmental categories (55%–59%) was all significantly higher than that of geographical distance (20%–23%). Therefore, we excluded Geography group in the subsequent analysis and focused on the Physicochemistry and Nutrient categories. Results from Mantel test and Spearman’s correlation indicated that three core trophic groups were significantly influenced by various environmental stressors within both the Physicochemistry and Nutrient categories (lines marked in red or blue) (Fig. 4A–D; Fig. S4). Within the three trophic modes, there was a certain degree of environmental preference. To be specific, they were more strongly influenced by factors in the Nutrient group during the summer, while they tended to be more affected by factors in the Physicochemistry group in winter (Fig. 4B and D). Within the same trophic mode, however, no specific preference was observed. They were simultaneously influenced by multiple stressors across seasons in varying degrees (Fig. 4A and D). Notably, temperature consistently had a significant impact (higher Mantel’s *r*) across different seasons on all three groups (*P* < .001). Based on these results and considering the complexity of identifying key factors across temporal scales, we applied a robust RF algorithm to explore the most important environmental stressors shaping the relative competitive advantage of core mixoplankton (Fig. 4E). Of the two environmental categories, half of the factors had a significant impact on core mixotroph abundance (*P* < .05). The ranking of the percentage of increase in MSE indicated that the influence of temperature, DO, Secchi, salinity, and pH in the Physicochemistry group decreased sequentially. In the Nutrient group, the subsequent influential factors were NO_3_-N and PO_4_-P (Fig. 4E). Therefore, the top three factors, temperature and DO in the Physicochemistry group and NO_3_-N in the Nutrient group, were identified as the dominant environmental stressors for core mixoplankton.

### Niche preferences of core mixoplankton across three stressors gradients

**Figure 5:**
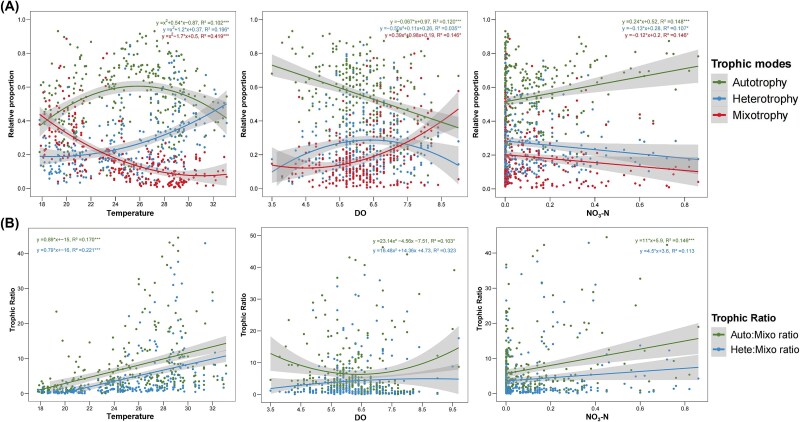
The environmental gradients of three key stressors show linear or nonlinear correlations with three core trophic taxa. The relative proportion (A–C) and trophic taxa ratio (D–F) of autotroph, heterotroph, and mixotroph covaried with temperature, DO and NO_3_-N (nitrate) gradient. Trophic ratio, used as an indicator of mixotroph competitiveness, is defined as the ratio of core autotrophs or core heterotrophs to core mixoplankton. A higher value indicates weaker mixotroph competitiveness. The corresponding equation, R square, and significance level are shown at the top of each figure. Significance levels: ^*^P < .05, ^*^^*^P < .01, and ^*^^*^^*^P < .001. The gray shade represents 95% confidence intervals.

The relative competitiveness of three core trophic groups was represented by their relative proportions and the pairwise ratios with mixoplankton (Fig. 5). The results showed that the three dominant stressors were generally significantly correlated, either nonlinearly or linearly, with both relative proportions and trophic ratios.

For relative proportion, three core trophic groups showed linear correlations with NO_3_-N (Fig. 5C), while the relationships with temperature and DO were generally nonlinear (Fig. 5A and B). Core mixoplankton exhibited a decreasing trend along both temperature and NO_3_-N gradients, while showing a nonlinear positive correlation with DO. Additionally, although core autotrophic taxa consistently maintained higher relative proportions across the gradients of all three stressors, core mixoplankton surpassed heterotrophs in relative abundance where the temperature was below 23°C or DO exceed 7.5 mg/L when considering a single factor (Fig. 5A and B). For trophic ratio, the pairwise ratios with core mixotroph group were linearly positively correlated with temperature and NO_3_-N gradients (Fig. 5D and F), but nonlinearly correlated with DO (Fig. 5E). It showed that the Auto:Mixo ratio declined linearly as temperature and NO_3_-N increase, whereas moderate DO concentrations favored core mixoplankton in competition with core autotrophs. In general, core mixoplankton preferentially occurred under conditions characterized by low temperature and NO_3_-N levels, and high DO concentrations when considering individual factors (Fig. 5).

### Interplays and the potential optimal threshold identification of core mixoplankton under multiple stressors

**Figure 6:**
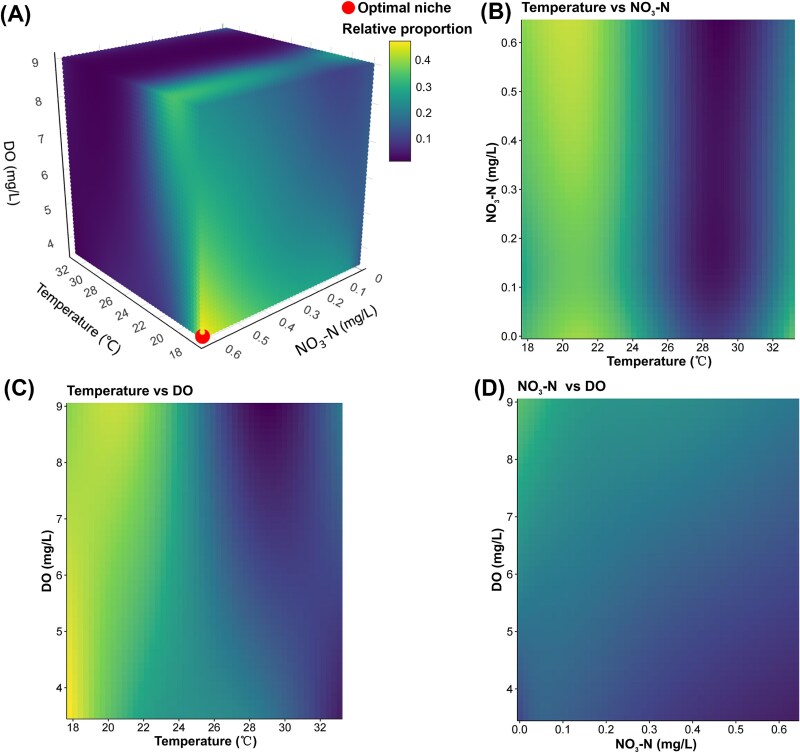
GAMM reveals interaction effects of three key stressors on the competitiveness of core mixoplankton. (A) Gradient changes in relative proportion of core mixoplankton are modeled and predicted at three-dimensional scales by simultaneously considering the effects of temperature, DO, and NO_3_-N and their interplays. The potential optimal niche indicates which predicted relative proportion is highest at that coordinate point. (B– D) The dynamics of core mixotrophic relative proportion in response to pairwise interactions among key stressors, including temperature *vs* NO_3_-N (B), temperature *vs* DO (C), NO_3_-N *vs* DO (D).

GAMM was employed to further examine the complex interactions of three key driving forces on the dynamics of mixoplankton. The interactive 3D plot demonstrated the combined effects of temperature, NO_3_-N, and DO on the predicted relative proportion of core mixoplankton (Fig. 6A). Their dynamics were represented in space with color gradients, indicating the niche preferences. Remarkably, a distinct point (17.8°C, 0.64 mg/L NO_3_-N and 3.5 mg/L DO) was identified where the predicted relative proportion of core mixoplankton reached the maximum, highlighting the optimal thresholds of multiple stressors (Fig. 6A).

Moreover, the pairwise interactions among three stressors were illustrated by 2D heatmaps (Fig. 6B–D). In general, core mixoplankton exhibited the most pronounced variations along the temperature gradient (Fig. 6B and C), showing a decreasing trend with increasing temperature, although a slight increase was observed at the high temperature (>30°C) (Fig. 6B). Nevertheless, NO_3_-N and DO exerted varying degrees of compound effects in shaping the competitive landscape of core mixoplankton. Specifically, while their relative proportion maintained relatively high at lower temperatures (18°C–24°C) (Fig. 6B), elevated NO_3_-N concentrations further enhanced their relative competitive advantage (deeper yellow coloration). In addition, the heatmap under temperature and DO gradients illustrated that although low levels of DO and temperature favored core mixoplankton, high DO concentrations helped counteract the declining trend in their relative proportion induced by increasing temperature (Fig. 6C).

## Discussion

Exploring how the three major trophic groups, particularly mixoplankton, gain dynamic relative competitive advantages under multiple environmental stressors remains challenging [5, 6, 33]. Our study focused on and emphasized the methods for identifying core trophic taxa and key stressors to bridge the gap between limited database resources and dynamic complexity. By further modeling the stressors using RF and GAMM, we aimed to uncover the potential optimal ecological niche for core mixoplankton and map their dynamic responses under interactive effects. This approach, adhering to the idea of dimensionality reduction under the influence of multiple stressors in the bioinformatics analysis, largely reduces the complexity and heterogeneity by representatively reflecting dynamic patterns of the whole community. It provides a new methodology to address the current low annotation efficiency and the identification of key factors in plankton community, while also offering insights for ecological hypotheses and simplifying dimensions prior to model construction.

### Selection of core taxa and three trophic modes annotation

Marine microbial communities include core taxa that are usually key for ecosystem function. Despite their importance, these core taxa are relatively unknown, reflecting a lack of consensus on how to identify them [23, 38]. To date, most core microbiomes have been defined based on species occurrence and abundance. However, it is necessary to integrate ecological insights with experimental design on different scales to determine the ecologically-supported core taxa and thresholds [39]. Here, we employed a robust method that uses abundance-occupancy distributions to prioritize core microbiomes over space and time [21]. This approach significantly reduces the number of species requiring trophic annotation while comprehensively assessing species ubiquity and their contribution to community structure. Our results showed that the core taxa that constituted a small proportion of total ASVs richness contributed to over 80% of β-diversity (Fig 1B and C, S2), highlighting their disproportionately significant role in shaping community diversity. Furthermore, it is challenging to qualitatively determine the immediate mode of mixoplankton because the trophic strategy is really a continuum between pure autotroph and heterotroph [3, 40]. Here, we adopted a commonly used approach combined database and manual curation to annotate the potential nutritional strategies across lineages. This method allows us to incorporate the latest research findings, overcoming potential lag in current databases. It ensures greater accuracy and timeliness, particularly in recognizing the ever-evolving mixotrophic taxa [12, 41]. Despite the potential for subjective bias, the approach is expected to remain the mainstream and relatively optimal solution for trophic annotation in plankton research at the community level in the future.

### Spatial distribution and seasonal patterns of the trophic groups

The high dynamics in environmental parameters suggested that the Hong Kong waters were strongly influenced by anthropogenic impacts or natural disturbances (Fig. S1). Although previous studies indicated that core taxa tend to exhibit relatively higher sensitivity and resilience in response to multiple environmental stressors [34, 42], diversity of three core groups displayed distinct seasonal and geographical patterns, reflecting their unique ecological strategies and potential adaptive mechanisms (Fig. 2). Despite the high local Shannon diversity, the core autotrophic communities demonstrate a high stability in α-diversity, providing fundamental resources for the community. The trait trade-offs characteristic of mixoplankton confers a buffering effect under rapid environmental changing and increases food web competition, thus offering a certain degree of competitive advantage [3, 5]. Beyond demonstrating clear seasonal patterns in abundance and α-diversity, our findings further revealed that the flexible resource acquisition strategies of core mixoplankton confer adaptive advantages during temporal turnover, allowing them to reach a peak in winter and exhibit higher spatial uniformity compared to core heterotrophs. (Fig. 1, Fig. 2). Additionally, the relatively consistent β-diversity patterns shared among the three core trophic groups can be attributed to differences in nutritional, physicochemical and geographical factors [33, 43, 44]. The VPA results further quantitatively determined the driving forces behind the significant seasonal variations, with Geography category contributing the least. It may stems from the high environmental heterogeneity and dense sampling distribution. This highlighted the dominant importance of Physicochemical and Nutrient categories on a regional scale (Fig. 3D–F). In contrast to current studies focusing on prevalence surveys at larger scales [6, 41], our study bridged the gap and emphasized the importance of core trophic groups, particularly core mixoplankton, in responding to seasonal patterns within this highly dynamic environment of subtropical coastal regions.

### Unraveling the key environmental drivers shaping the relative success of core mixoplankton

Amid escalating climate change and intensifying human activities, coastal ecosystems are expected to experience disturbances from multiple environmental stressors, including warming, eutrophication, and hypoxia [11, 16, 43]. We identified complex cross-seasonal interactions between 14 environmental factors and three trophic groups by Mental test and Spearman’s correlation (Fig. 4A–D). This complexity may result from the highly fluctuating environmental pressures in Hong Kong waters [30], or from the ability of these trophic groups to adjust their niche preferences according to seasonal conditions and resource availability [5, 45]. For instance, the higher abundance and diversity of core mixoplankton observed in spring and winter were more influenced by physicochemical factors than nutrients (Fig. 4D). It may be attributed to the lower nutrient and temperature levels, and high physicochemical variations during wet seasons (Fig. S1). Although the general influence of temperature on the growth and metabolism of plankton communities was well-documented, most studies focused on the trade-offs and shifts from mixotrophy to heterotrophy in response to reduced photosynthesis, overlooking the impact on the abundance and relative proportion of mixoplankton themselves [10, 18, 46]. Our findings highlighted that temperature served as a key environmental stressor for all three trophic groups across seasonal scales (Fig. 4A–D). Furthermore, the relative proportion and α diversity of core mixotrophic taxa decreased significantly as the temperature rises (Fig. 5A and D), reflecting their unique adaptability during colder seasons. In addition, recent studies have examined the linear or nonlinear effects of individual environmental factors on mixoplankton [41, 43, 44]. Similar to core taxa, the overall prevalence of mixoplankton is negatively correlated with nutrient concentrations. And their relative success in oligotrophic waters and seasons is due to a generalist trophic strategy that has been confirmed across various scales and models [7, 47]. In addition, a few studies explored the importance of DO as a key driving force. Zou *et al.* [44] identified DO as a critical and independent factor across three functional traits and demonstrated its significant positive linear relationship with mixoplankton, which basically aligned with our findings (Fig. 5A). However, Wang *et al.* [43] discussed the complex causality, suggesting that high temperature, eutrophication, and reduced light availability could lead to lower DO levels. It further emphasized the limitations of considering the single linear and / or nonlinear interactions.

### Multiple stressors shape the relative competitive advantage of core mixoplankton

Coastal waters under global change and anthropogenic disturbances are commonly subjected to multiple stressors, yet their compound ecological effects are rarely explored [15, 29]. In contrast to the single-factor analyses in Fig. 5, the potential optimal niche identified by GAMM indicated that high NO_3_-N levels, along with low temperature and DO levels, led to the greatest relative success of core mixoplankton (Fig. 6A). It indicated that when considering combined effects, the optimal threshold for NO_3_-N and DO were adjusted upward and downward, respectively, while the temperature remains constant. The finding highlighted potential bias in discussing the factors separately and the necessity of considering multiple environmental stressors simultaneously [16, 29]. Additionally, similar to the results based on controlled experiments and field observations [10, 25], temperature exhibited the strongest impact among three stressors in the 2D heatmap (Fig. 6B and C), while the effect of NO_3_-N was weakened due to potential antagonistic interactions with temperature gradient. This may result from elevated temperatures shifting mixoplankton toward heterotrophy, reducing their sensitivity to NO_3_-N gradient [18, 48]. Core mixoplankton generally favored environments with low temperatures and high NO_3_-N levels, aligning with the optimal niche result (Fig. 6A and B). In contrast, DO distinctly extended the temperature tolerance range for core mixoplankton, indicating potential synergistic or additive effects. However, current modeling studies have largely overlooked these compound effects and underestimated their direct or indirect influence [6, 41]. For example, Edwards [7] used a GAMM with individual predictors and found that mixoplankton were uncorrelated to temperature due to data limitations, instead driving the dynamics of the three trophic groups by higher irradiance at lower latitudes. Overall, our relatively comprehensive modeling approach offers detailed insights into the responses of trophic functional groups to multiple stressors. These results reinforced the complex interactions among key stressors in shaping the competitive landscape of mixoplankton, rather than simply considering the additive effects of individual parameters.

## Conclusion

In conclusion, this study was conducted using high-density spatial and time-series sampling in Hong Kong waters. By identifying core trophic groups and systematically uncovering key stressors, we employed GAMM to decipher and highlight how complex interplays shaped the relative success of core mixoplankton. The findings highlighted the strong impact of temperature and potential bias in discussing the factors separately. Our approach could also be extended to explore the spatiotemporal dynamics of the whole trophic functional communities, providing insights into tracking and predicting the long-term trajectories and multi-dimensional responses. In the future, the combined efforts of theoretical ecology, empirical and modeling research will enhance our understanding of the dynamic competition of mixoplankton to changing environments.

## Supplementary Material

FS1_ycaf053

FS2_ycaf053

FS3_ycaf053

FS4_ycaf053

FS5_ycaf053

FS6_ycaf053

TableS1_ycaf053_revised

TableS2_ycaf053

TableS3_ycaf053_revised

Supplementary_figure_and_legends_ycaf053

Supplementary_table_legends_ycaf053

Supplementary_text_ycaf053

## Data Availability

The datasets presented in this study can be found in online repository Genome Sequence Archive (GSA) with accession number CRA022002.
