# Supplementary figures and images for: Deciphering the key stressors shaping the relative success of core mixoplankton across spatiotemporal scales
Source: ISME Commun. 2025 Mar 26;5(1):ycaf053. doi: 10.1093/ismeco/ycaf053 (PMC12017963; doi:10.1093/ismeco/ycaf053)

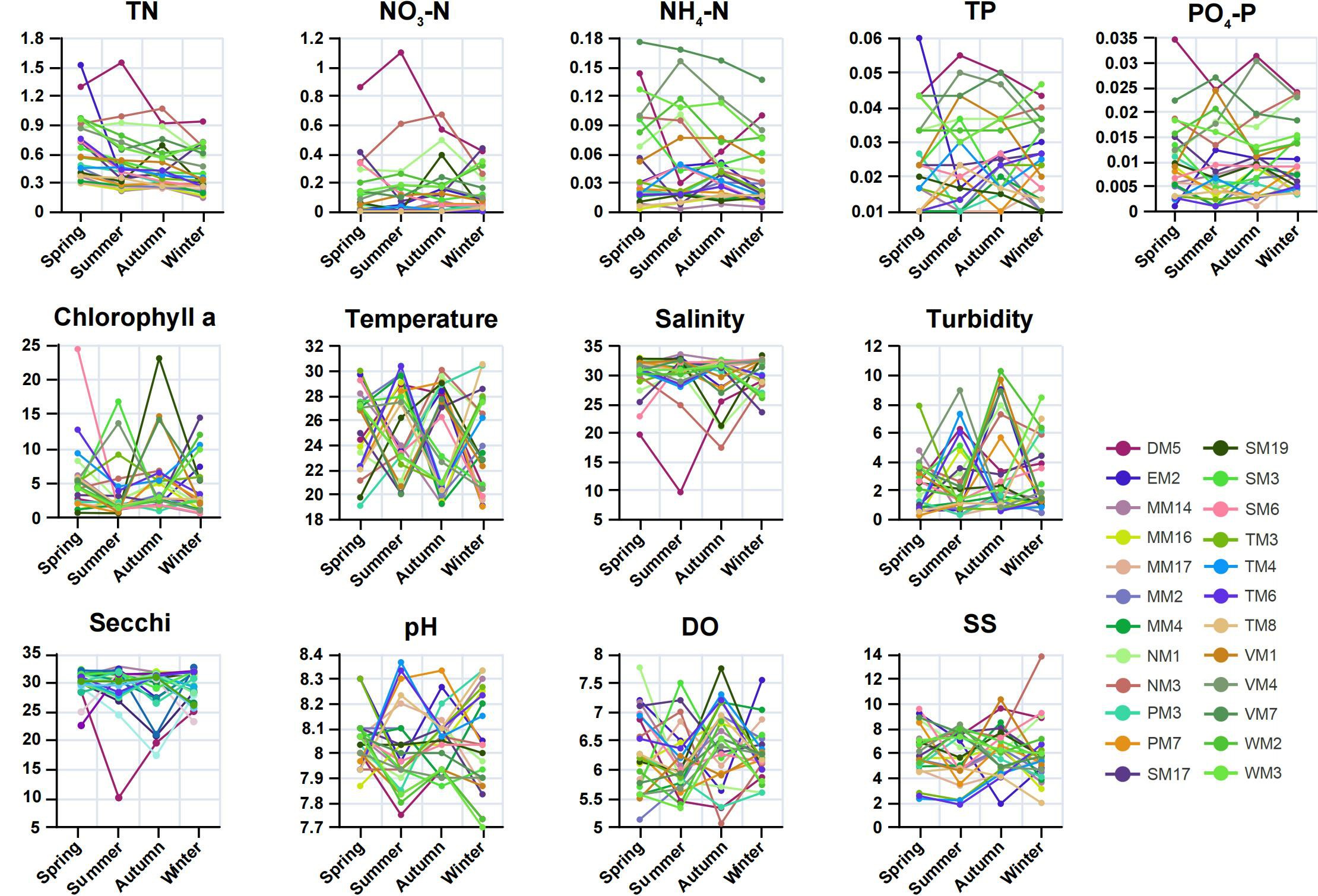

Supplement: FS1_ycaf053 [file fs1_ycaf053.jpeg]

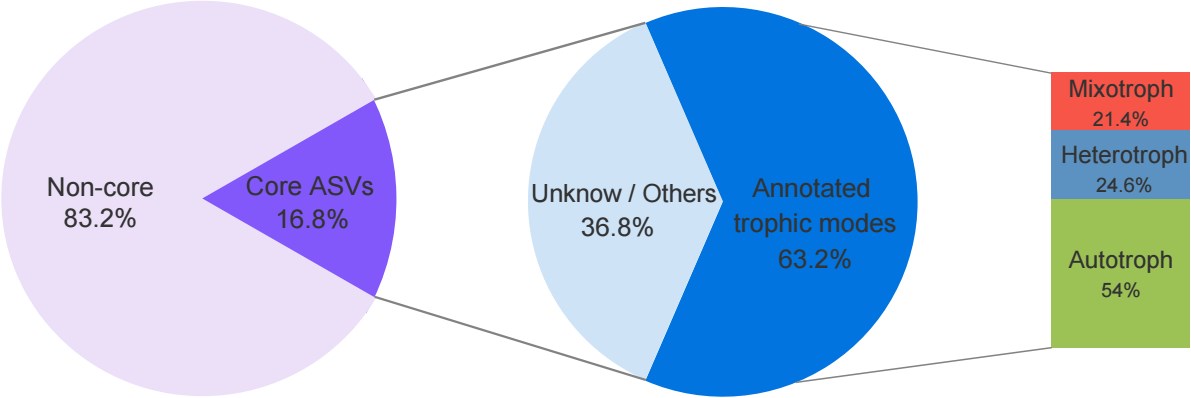

Supplement: FS2_ycaf053 [file fs2_ycaf053.jpeg]

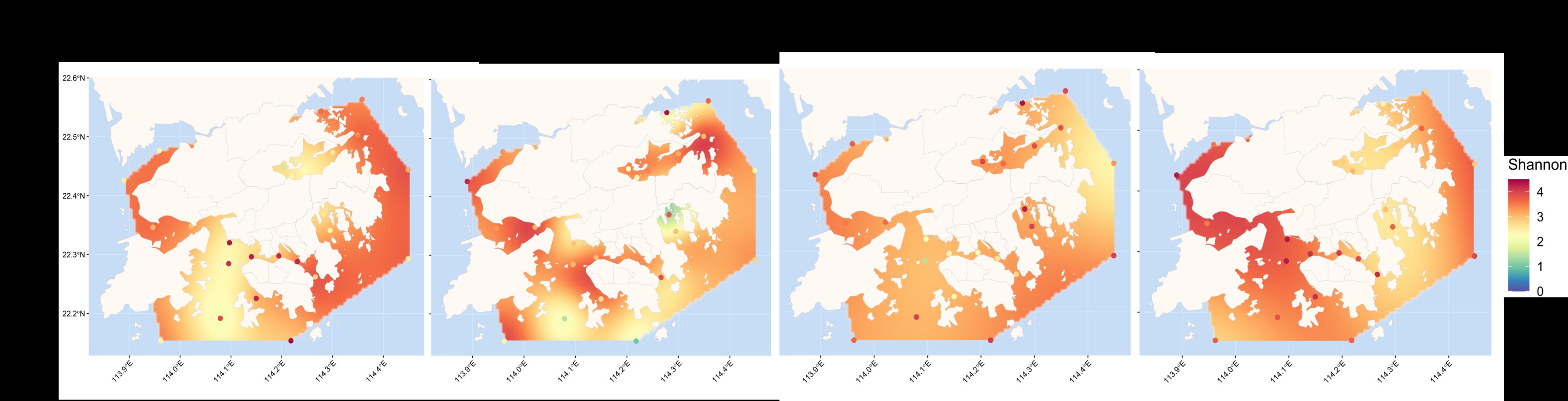

Supplement: FS3_ycaf053 [file fs3_ycaf053.jpeg]

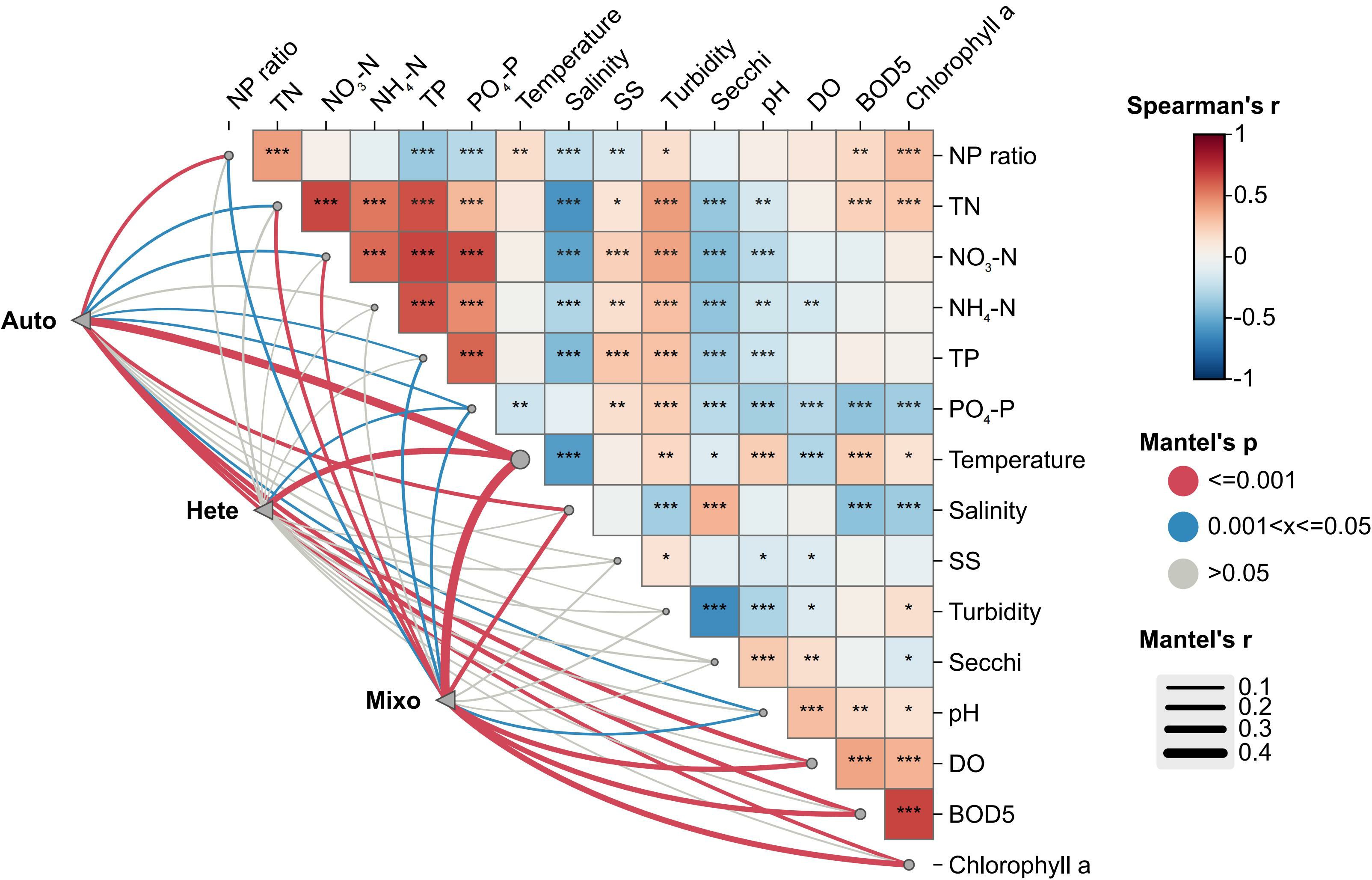

Supplement: FS4_ycaf053 [file fs4_ycaf053.jpeg]

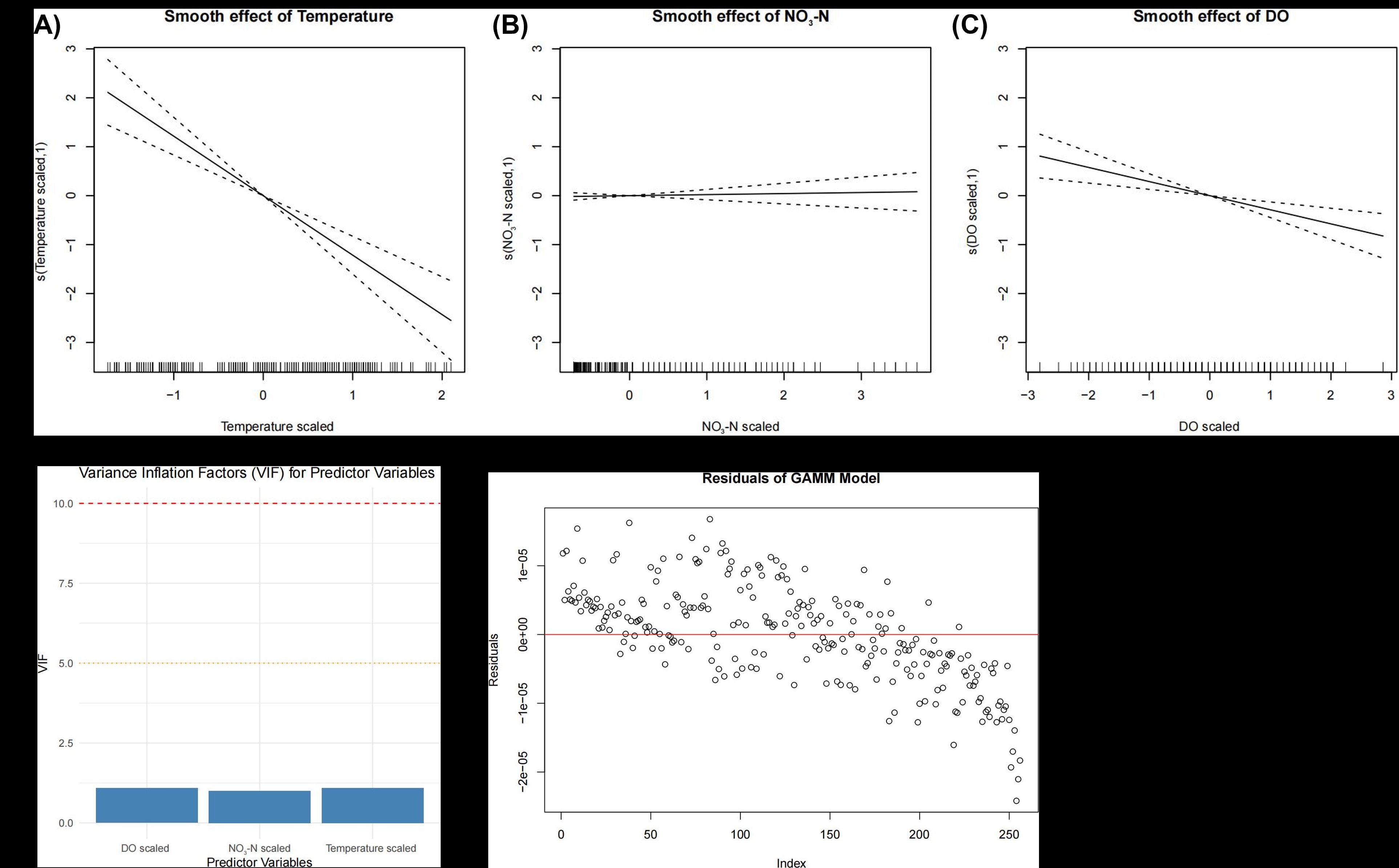

Supplement: FS5_ycaf053 [file fs5_ycaf053.jpeg]

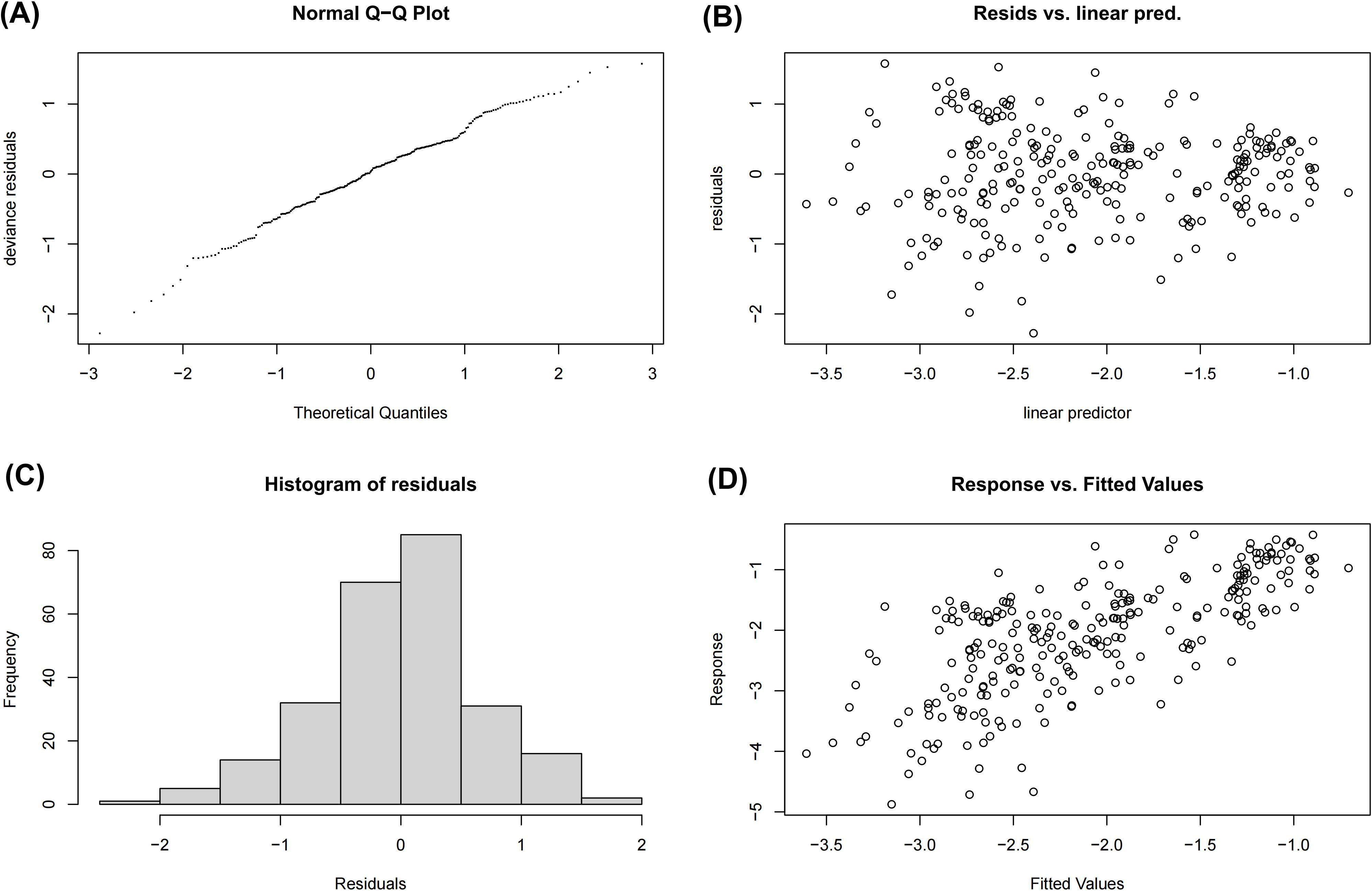

Supplement: FS6_ycaf053 [file fs6_ycaf053.jpeg]
