# Supplementary material for: Deciphering the key stressors shaping the relative success of core mixoplankton across spatiotemporal scales
Source: ISME Commun. 2025 Mar 26;5(1):ycaf053. doi: 10.1093/ismeco/ycaf053 (PMC12017963; doi:10.1093/ismeco/ycaf053)
Supplement: Supplementary_figure_and_legends_ycaf053 [file supplementary_figure_and_legends_ycaf053.docx]

Supporting information to
“Deciphering the key stressors shaping the relative success of core mixoplankton across spatiotemporal scales”

Zhicheng Ju^1^, Sangwook Scott LEE^1^, Jiawei Chen^1^, Lixia Deng^1^, Xiaodong Zhang^1^, Zhimeng Xu^1^, Hongbin Liu^1, 2*^

^1^Department of Ocean Science, The Hong Kong University of Science and Technology, Hong Kong SAR, China

^2^ Hong Kong Branch of Southern Marine Science and Engineering Guangdong Laboratory (Guangzhou), Hong Kong SAR, China

***** Corresponding Author:

Hongbin Liu, Department of Ocean Science, Hong Kong University of Science and Technology, Clear Water Bay, Hong Kong, 000000, China. E-mail: [liuhb@ust.hk](mailto:liuhb@ust.hk)

## Supplementary Figures S1 to S6 attached to the main text


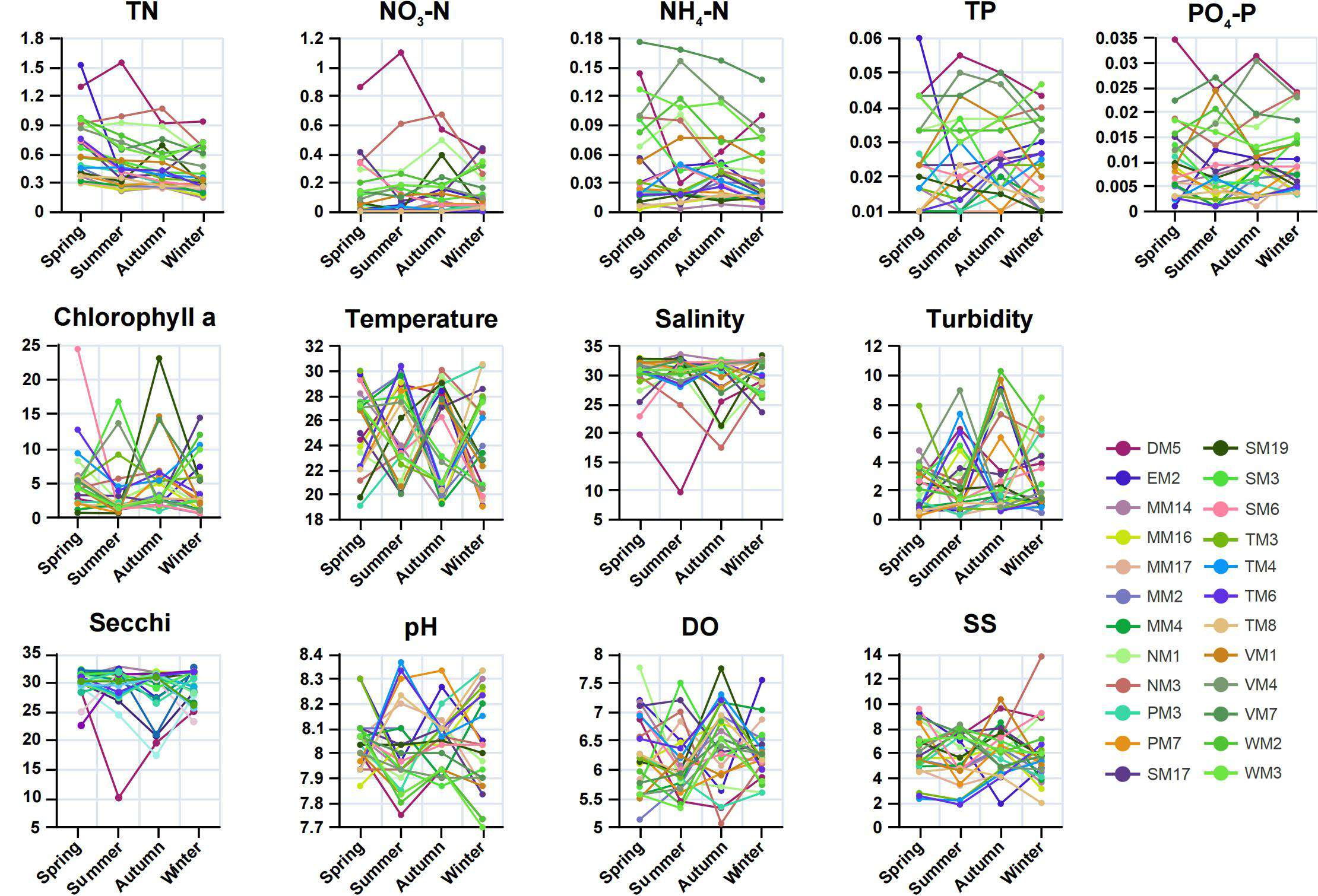


### **Figure S1.** **Seasonal dynamics of 13 environmental factors at 24 sampling sites.**

13 environmental factors are TN, total nitrogen; NO_3_-N, nitrate; NH_4_-N, ammonium; TP, total phosphorus; PO_4_-P, phosphate phosphorus; Chlorophyll a; temperature; salinity; Turbidity; Secchi; pH; DO, dissolved oxygen; SS, suspended solids. Each color in the line chart legend represents a sampling site.

**
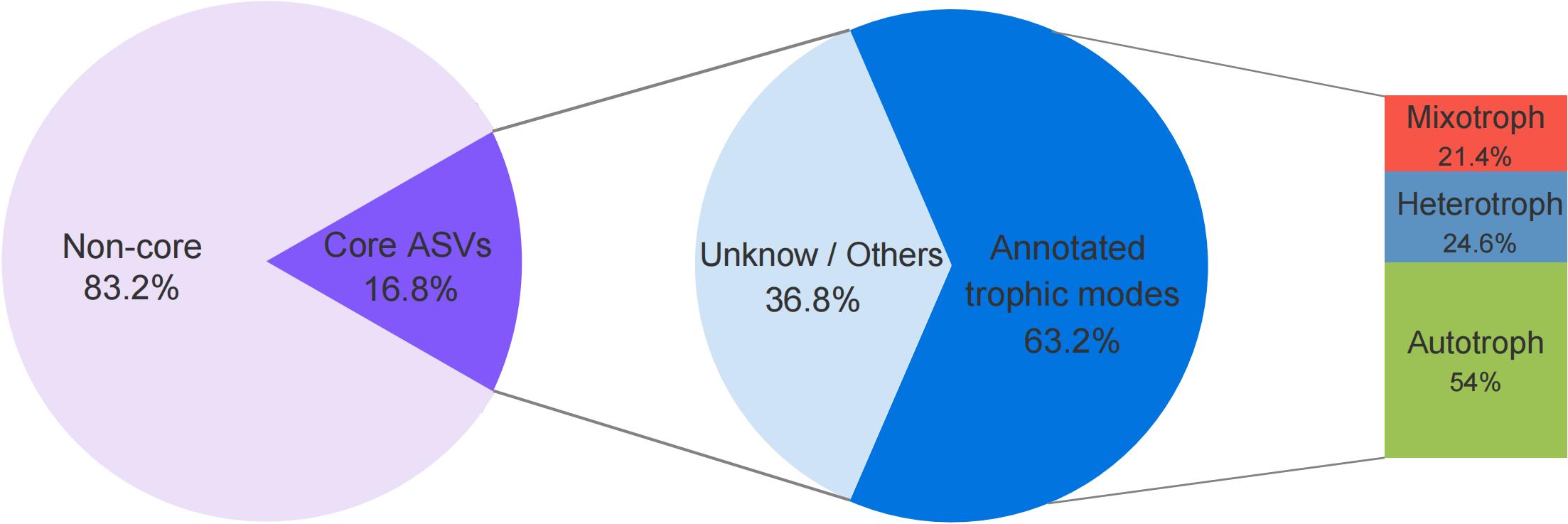
**

### Figure S2. Percentages of core ASVs selected and their trophic annotation.

Based on the Method described, 16.8% of amplicon sequence variants (ASVs) were identified as core taxa (912 ASVs). Subsequently, 63.2% of them were successfully assigned to three trophic modes (Autotroph, Heterotroph and Mixotroph) by combining the database and manual curation.

**
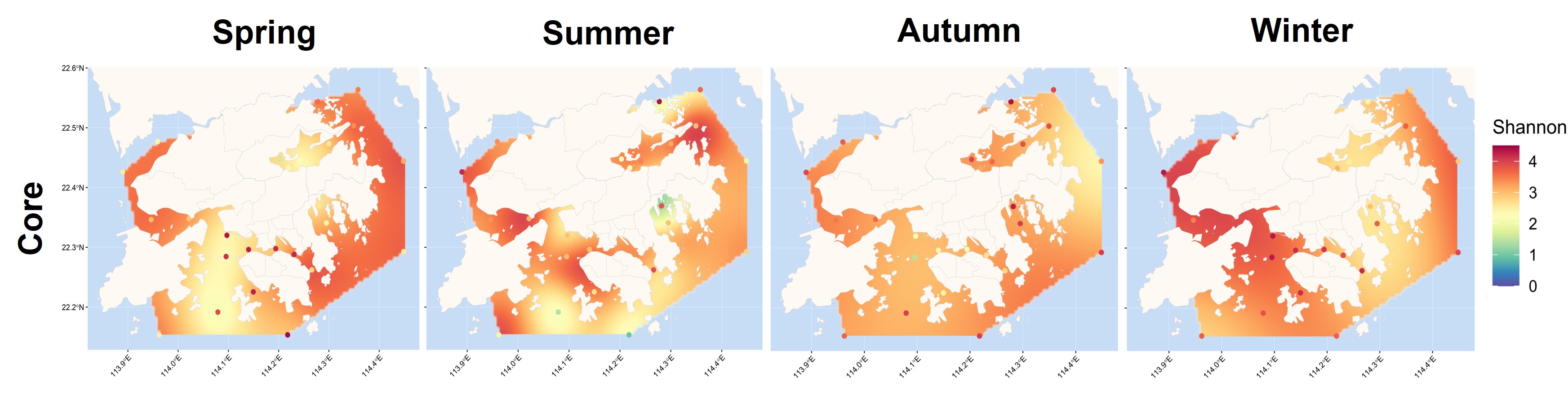
**

### Figure S3. Predicted spatiotemporal distribution of core taxa α-diversity (Shannon index) using the kriging interpolation method.


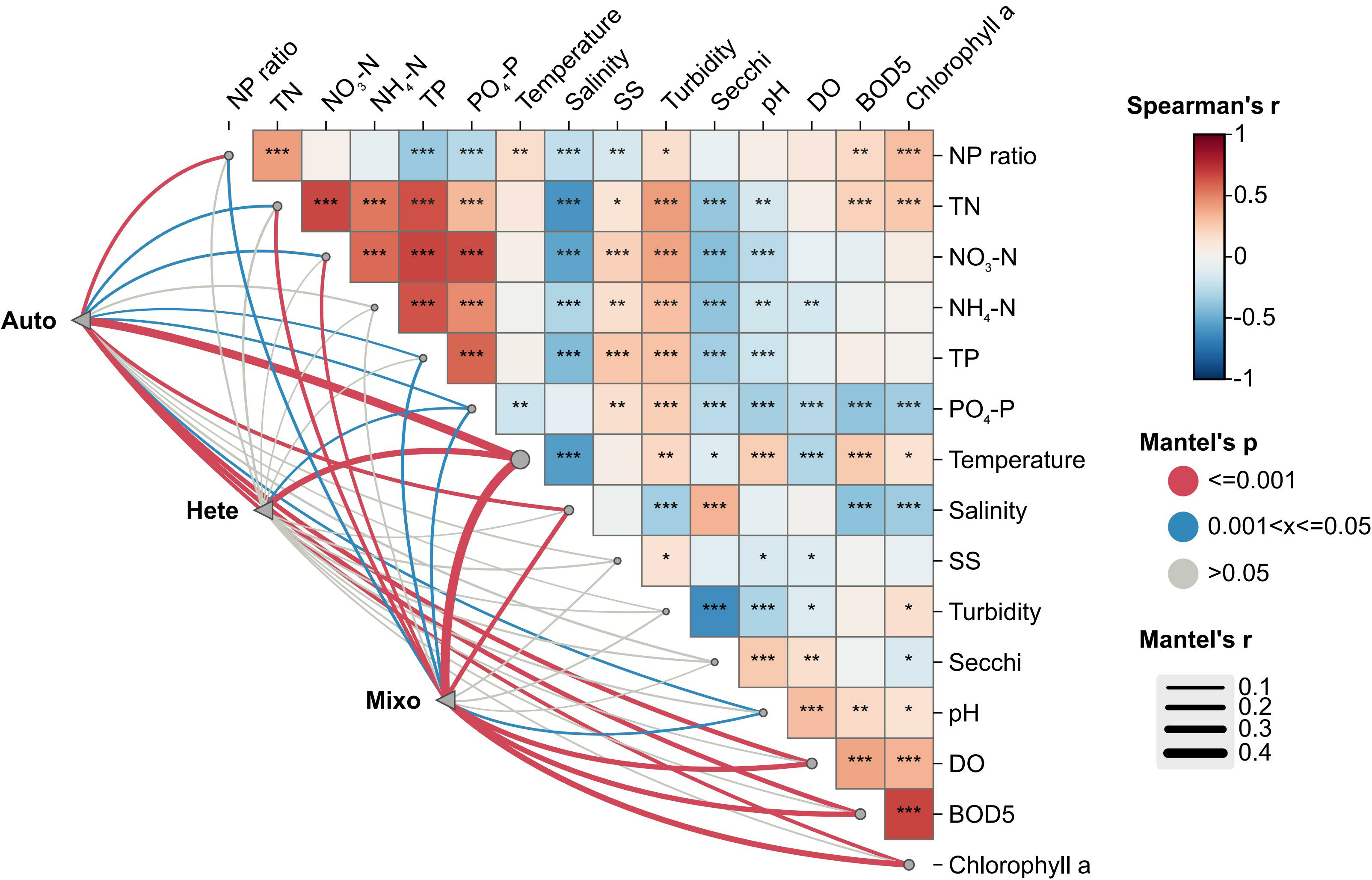


### Figure S4. Seasonal average of correlation between environmental factors and three trophic groups.

Pairwise comparisons of 15 environmental factors are shown at the upper-right, with a color gradient representing Spearman’s correlation coefficients. *** indicates *p*< 0.001; ** indicates *p* < 0.01; * indicates *p* < 0.05. Three trophic taxa were correlated to each factor by partial Mantel test. The line width represents the partial Mantel’s r statistic for the corresponding correlation, and line color means that significances are tested based on 999 permutations. NP ratio, total nitrogen: total phosphorus ratio; TN, total nitrogen; NO_3_-N, nitrate; NH_4_-N, ammonium; TP, total phosphorus; PO_4_-P, phosphate phosphorus; temperature; salinity; SS, suspended solids; Turbidity; Secchi; pH; DO, dissolved oxygen; BOD5, five-day biochemical oxygen demand; Chlorophyll a.


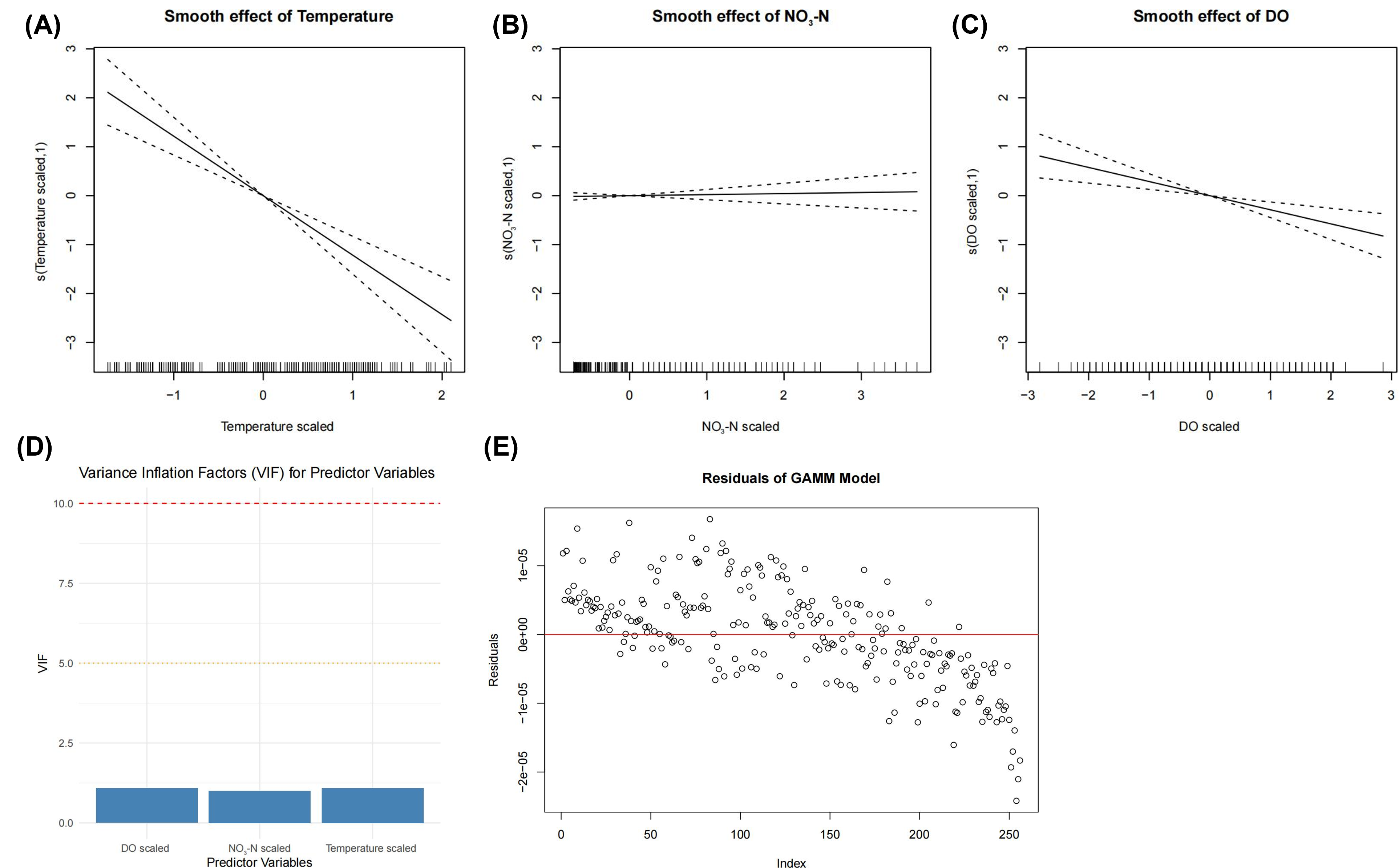


### Figure S5. Smooth effect, variance Inflation Factors (VlF), and residual plots for the three environmental drivers in Generalized Additive Mixed Model (GAMM).

(A-C) Smooth effects of temperature, nitrate (NO_3_-N), dissolved oxygen (DO) on mixotrophic reads. The solid line represents the fitted smooth term, and the dashed lines indicate the 95% confidence intervals. (D) The yellow and red dashed lines indicate VIF thresholds of 5 and 10, respectively, representing moderate and severe multicollinearity. VIF values below 5 suggest acceptable levels of correlation among predictors. (E) Residuals of the GAMM model. Each point represents the residual of an individual observation, defined as the difference between the observed value and the model-predicted value. The red horizontal line denotes the zero-residual line.


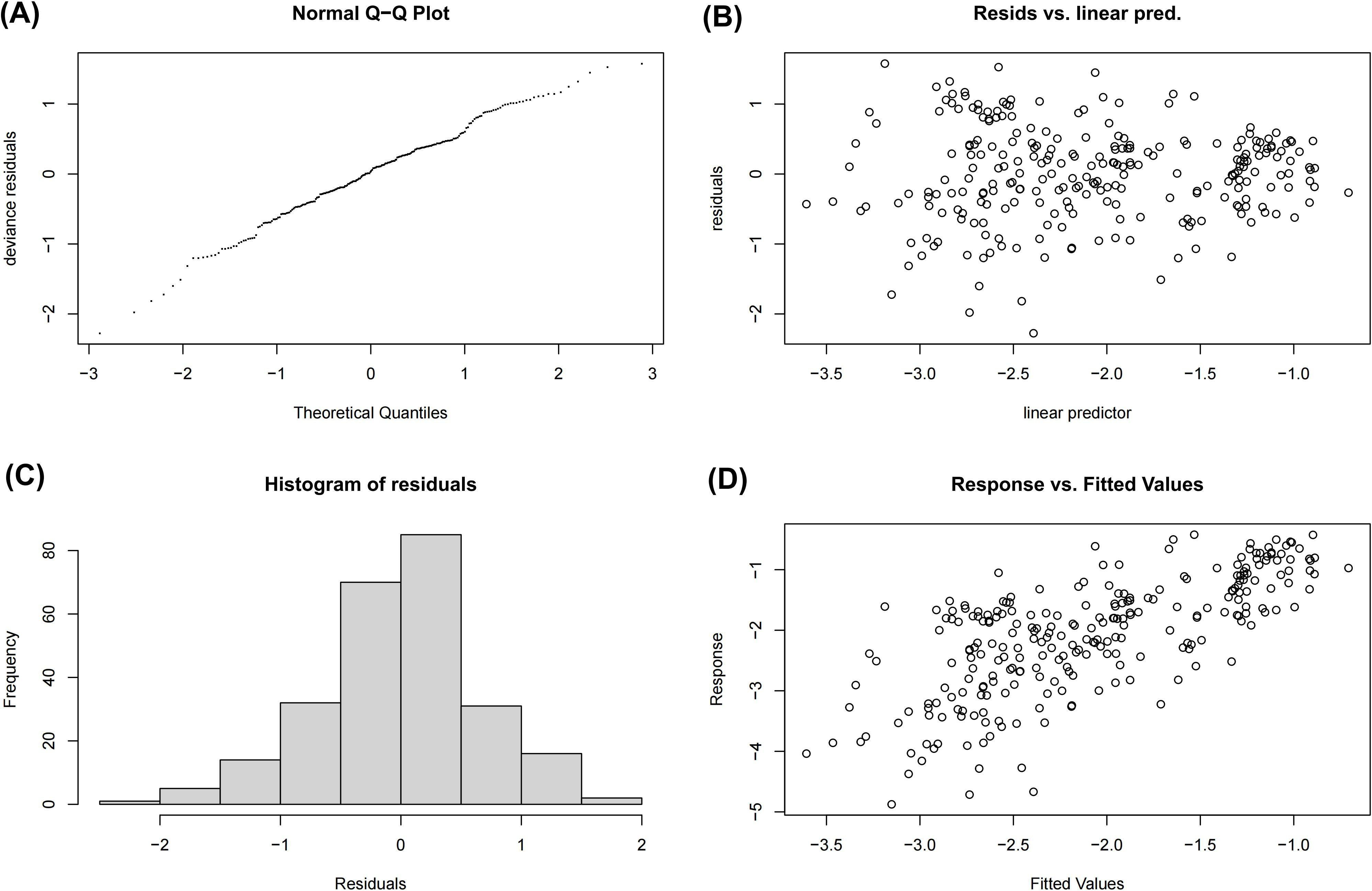


### Figure. S6 Diagnostic plots for assessing model assumptions and fit.

1. Normal Q-Q plot of deviance residuals, showing the relationship between theoretical quantiles and sample quantiles to evaluate the normality of residuals. (B) Residuals vs. linear predictor plot, displaying the distribution of residuals across the range of predicted values to check for homoscedasticity and linearity. (C) Histogram of residuals, illustrating the frequency distribution of model residuals to assess their overall shape and symmetry. (D) Response *vs*. Fitted Values plot, depicting the relationship between observed responses and model-predicted values to evaluate the model's predictive performance and identify potential patterns or outliers.
