# Supplementary material for: Deciphering the key stressors shaping the relative success of core mixoplankton across spatiotemporal scales
Source: ISME Commun. 2025 Mar 26;5(1):ycaf053. doi: 10.1093/ismeco/ycaf053 (PMC12017963; doi:10.1093/ismeco/ycaf053)
Supplement: Supplementary_table_legends_ycaf053 [file supplementary_table_legends_ycaf053.docx]

Supporting information to
“Deciphering the key stressors shaping the relative success of core mixoplankton across spatiotemporal scales”

Zhicheng Ju^1^, Sangwook Scott LEE^1^, Jiawei Chen^1^, Lixia Deng^1^, Xiaodong Zhang^1^, Zhimeng Xu^1^, Hongbin Liu^1, 2*^

^1^Department of Ocean Science, The Hong Kong University of Science and Technology, Hong Kong SAR, China

^2^ Hong Kong Branch of Southern Marine Science and Engineering Guangdong Laboratory (Guangzhou), Hong Kong SAR, China

***** Corresponding Author:

Hongbin Liu, Department of Ocean Science, Hong Kong University of Science and Technology, Clear Water Bay, Hong Kong, 000000, China. E-mail: [liuhb@ust.hk](mailto:liuhb@ust.hk)

**Supplementary Tables S1 to S3 attached to the main text**

Table S1: Taxonomy and trophic mode annotation of core set.

Table S2: Three groups of Environmental factors for variance decomposition analysis.

Table S3: Abstract of Generalized Additive Mixed Model.
