# Supplementary material for: Deciphering the key stressors shaping the relative success of core mixoplankton across spatiotemporal scales
Source: ISME Commun. 2025 Mar 26;5(1):ycaf053. doi: 10.1093/ismeco/ycaf053 (PMC12017963; doi:10.1093/ismeco/ycaf053)
Supplement: Supplementary_text_ycaf053 [file supplementary_text_ycaf053.docx]

Supporting information to
“Deciphering the key stressors shaping the relative success of core mixoplankton across spatiotemporal scales”

Zhicheng Ju^1^, Sangwook Scott LEE^1^, Jiawei Chen^1^, Lixia Deng^1^, Xiaodong Zhang^1^, Zhimeng Xu^1^, Hongbin Liu^1, 2*^

^1^Department of Ocean Science, The Hong Kong University of Science and Technology, Hong Kong SAR, China

^2^ Hong Kong Branch of Southern Marine Science and Engineering Guangdong Laboratory (Guangzhou), Hong Kong SAR, China

***** Corresponding Author:

Hongbin Liu, Department of Ocean Science, Hong Kong University of Science and Technology, Clear Water Bay, Hong Kong, 000000, China. E-mail: [liuhb@ust.hk](mailto:liuhb@ust.hk)

## Supplementary text for Generalized Additive Mixed Model

The four Generalized Additive Mixed Model (GAMM) analyses were conducted with progressive complexity:

**1. Base Model**: The first model included the smooth terms for each of the standardized environmental variables, where s () denotes a smooth function estimated using cubic regression splines. It also included a random effect for sampling stations (Sample), accounting for spatial variation.

**Base Model <- gamm (log_Relative_proportion ~ s (Temperature_scaled) + s** **(NO_3__N_scaled) + s (DO_scaled),**

**family = gaussian (), random = list (Sample = ~1), data = data)**

**2. Interaction Model 1**: To capture potential interactions between temperature and NO_3_-N, we then introduced a tensor product spline (te ()) to model the two-dimensional interaction between these variables:

**Interaction Model 1 <- gamm (log_Relative_proportion ~ s (Temperature_scaled) + s (NO_3__N_scaled) + s (DO_scaled) + te (Temperature_scaled, NO_3__N_scaled), family = gaussian (), random = list (Sample = ~1), data = data)**

**3. Interaction Model 2:** The model further explored the interaction by adding another tensor product spline to account for the three-way interaction among temperature, NO_3_-N, and DO:

**Interaction Model 2 <- gamm (log_Relative_proportion ~ s (Temperature_scaled) + s (NO_3__N_scaled) + s (DO_scaled) + te (Temperature_scaled,** **NO_3__N_scaled) + te (Temperature_scaled, DO_scaled), family = gaussian (), random = list (Sample = ~1), data = data)**

**4. Interaction Model 3**: All possible two-way interactions were included using tensor product splines (te ()), with the aim of investigating how each pair among the three key stressors jointly affects relative proportion of core mixoplankton.

**Interaction Model 3 <- gamm (log_Relative_proportion ~ s (Temperature_scaled) + s (NO_3__N_scaled) + s (DO_scaled) + te (Temperature_scaled, NO_3__N_scaled) + te (Temperature_scaled, DO_scaled) + te (NO_3__N_scaled, DO_scaled), family = gaussian (), random = list (Sample = ~1), data = data)**

Since Interaction Model 3 had the lowest AIC value among all the models (Table S3), it was selected as the final model. The model's reliability was confirmed through diagnostic checks (Figs S5 and S6). The smooth effect plots exhibited significant non-linear relationships (Fig S5 a-c), with variance inflation factors (VIFs) all below 5 (Fig S5 d), indicating acceptable multicollinearity levels. Moreover, residuals were normally distributed and showed no patterns of bias, affirming the robustness of the model's predictions (Fig S5 e). The diagnostic plots further confirmed the robustness of the GAMM (Fig S6). The Normal Q-Q plot showed that the residuals aligned well with the theoretical quantiles, indicating normality. The Residuals *vs*. Linear Predictor plot suggested that the model assumptions of homoscedasticity and linearity were met. Additionally, the Histogram of Residuals displayed a symmetrical distribution, reinforcing the model's accuracy. The Response *vs.* Fitted Values plot demonstrated a strong correlation between observed and predicted values, validating the model's predictive performance and overall fit.
